# Abundance of TRAIL attenuated by HIF2α and c-FLIP affects malignancy in renal cell carcinomas

**DOI:** 10.18632/oncotarget.25214

**Published:** 2018-05-01

**Authors:** Takahiro Isono, Tokuhiro Chano, Tetsuya Yoshida, Susumu Kageyama, Akihiro Kawauchi, Junji Yonese, Takeshi Yuasa

**Affiliations:** ^1^ Central Research Laboratory, Shiga University of Medical Science, Otsu, Shiga 520-2192, Japan; ^2^ Department of Clinical Laboratory Medicine, Shiga University of Medical Science, Otsu, Shiga 520-2192, Japan; ^3^ Department of Urology, Shiga University of Medical Science, Otsu, Shiga 520-2192, Japan; ^4^ Department of Urology, Cancer Institute Hospital, Japanese Foundation for Cancer Research, Tokyo 135-8550, Japan

**Keywords:** tumor necrosis factor related apoptosis-induced ligand, hypoxia inducible factor 2-alpha, cellular FLICE (FADD-like IL-1 beta-converting enzyme)-inhibitory protein, renal cell carcinomas, apoptosis

## Abstract

Dormant cancer cells are starvation-resistant leading to problems in the management of cancer. In renal cell carcinomas (RCCs), starvation-resistant cells are resistant to various currently available therapies. However, targeting hypoxia inducible factor 2-alpha (HIF2-alpha) induces cell death in dormant-like/starvation-resistant RCCs. This study showed that the apoptotic cell death caused by tumor necrosis factor (TNF)-related apoptosis-induced ligand (*TNFSF10*/TRAIL) was attenuated by CASP8 and FADD-like apoptosis regulator (*CFLAR*/c-FLIP) following HIF2-alpha activation, despite the high expression of TRAIL in such RCCs. Knockdowns of TRAIL averted apoptotic cell death caused by HIF2-alpha inhibition in starvation-resistant RCCs. Knockdowns of both HIF2-alpha and c-FLIP augmented apoptotic cell death, whereas overexpression of c-FLIP completely averted apoptosis. In addition, high abundance of TRAIL was correlated with poor prognosis in patients with RCC, suggesting that TRAIL, followed by HIF2-alpha and c-FLIP, play a role in the survival and/or progression of malignant RCCs.

## INTRODUCTION

Renal cell carcinoma (RCC) is the urological malignancy with the highest rate of mortality and an increasing incidence worldwide [1]. Clear cell RCC (ccRCC) is the most common form of RCC [2]. Generally, ccRCCs carry genetic insufficiencies of the von Hippel-Lindau tumor suppressor (VHL) and have shown constitutive activation of the hypoxia inducible factors (HIFs). It was previously reported that RCCs expressing HIF2-alpha rather than HIF1-alpha were malignant [3], and that the knockdown of HIF2-alpha induced cell death and/or inhibition of cell growth in RCCs both *in vitro* and *in vivo* [3–6].

Previous studies have classified RCC cells into two types, namely starvation-sensitive and starvation-resistant. Under glucose deprivation, starvation-sensitive RCCs produce *N*-linked (ß-*N*-acetylglucosamine)_2_ [*N*-GlcNAc_2_]-modified glycoproteins [7, 8]. These glycoproteins promoted cell death via unfolded protein responses in the endoplasmic reticulum [7, 9]. In contrast, starvation-resistant RCCs tolerated the deprivation condition without cytotoxic unfolded protein responses [7], and survived using their enhanced mitochondrial performance and sources of stored carbons [10]. The behavior of the latter type is similar to that of dormant cancer cells, which are often problematic during the therapeutic management of RCC. Interestingly, targeting HIF2-alpha induces cell death in the latter dormant-like RCC [11] (Supplementary Table 1).

This study analyzed the mechanism of cell death caused by inhibition of HIF2-alpha in dormant-like/starvation-resistant RCC. The results demonstrated that cell death was induced by tumor necrosis factor (TNF)-related apoptosis-induced ligand (TRAIL) (*TNFSF10*) [12], and TRAIL-mediated apoptosis was attenuated by HIF2-alpha and c-FLIP. The present study also indicated that higher abundance of TRAIL may be correlated with poor prognosis and has a prognostic value in patients with RCC.

## RESULTS

### TRAIL *(TNFSF10)* is highly expressed in starvation-resistant RCCs

**Figure 1:**
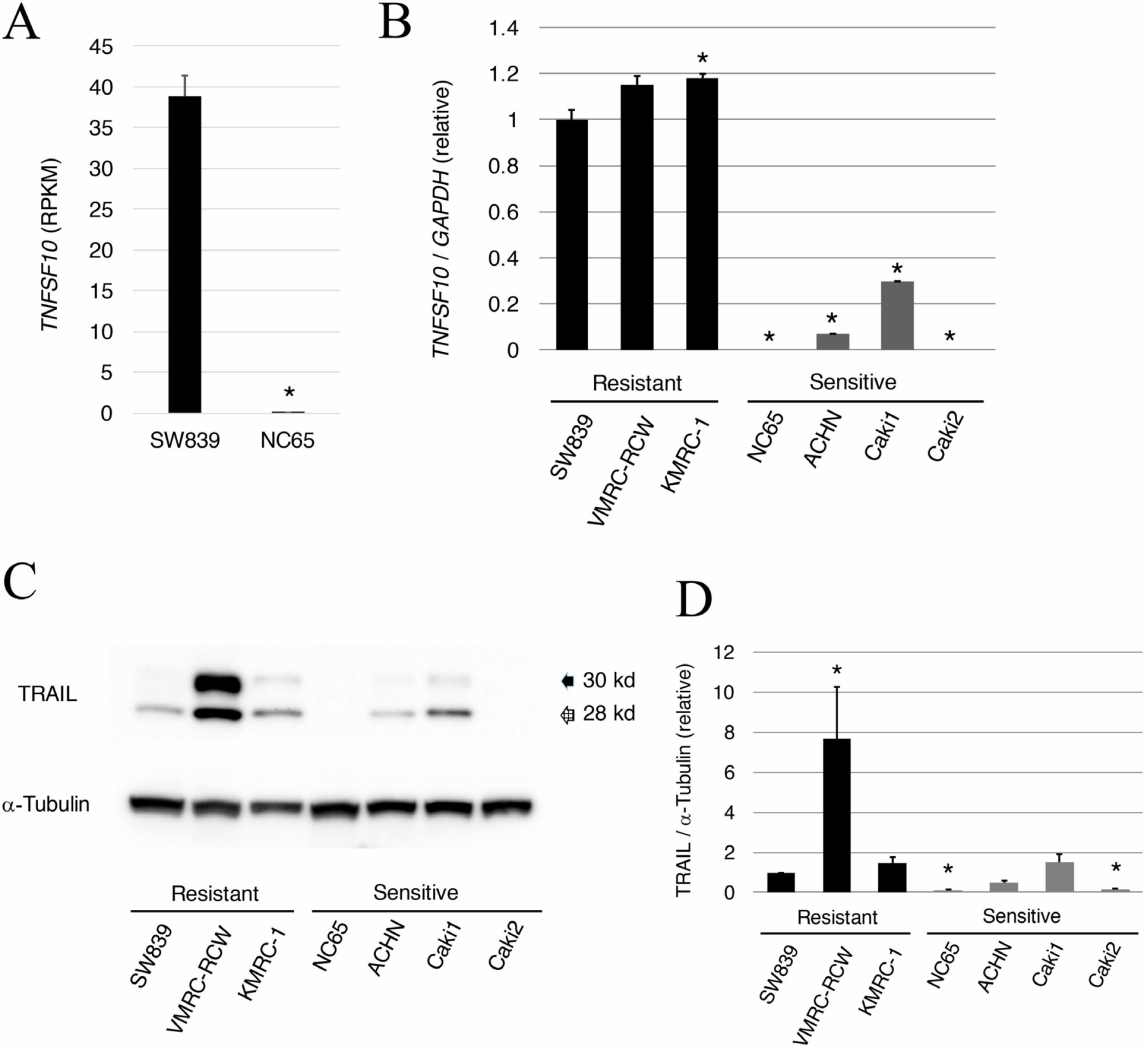
Abundant expression of TRAIL in starvation-ressistant renal cell carcinoma (**A**) RPKM value of *TNFSF10* (TRAIL) in the starvation-resistant SW839 RCC cell line and the starvation-sensitive NC65 RCC cell line. (**B**) Quantitative RT-PCR of TRAIL in the starvation-resistant (SW839, VMRC-RCW, and KMRC-1) and starvation-sensitive (NC65, ACHN, Caki1, and Caki2) RCC cell lines. (**C**) Data shown in panel B for TRAIL were confirmed by western blotting. (**D**) Quantitative graphic representation of data shown in panel C. Transcriptional and protein expressions were normalized against *GAPDH* and α-tubulin, respectively. Error bars represent standard errors from three independent experiments. ANOVA: F (6, 14) = 336.47, *p* = 2.614e^–14^; *p* < 0.05, pairwise comparisons using *t* tests with pooled SD vs. SW839 (^*^). Note that TRAIL mRNA and protein expression was higher in starvation-resistant cells than in starvation-sensitive cells.

Global transcriptional data of SW839 versus NC65, which are typical starvation-resistant and starvation-sensitive RCC cell lines, respectively, were investigated to clarify the mechanism of cell death induced by inhibition of HIF2-alpha in dormant-like/starvation-resistant RCC. The results showed that TRAIL (*TNFSF10*) was highly expressed in SW839 unlike NC65 cells (Figure 1A). TRAIL is a death ligand inducing apoptosis in tumor cells [12]. The expression of *TNFSF10* was re-evaluated in three starvation-resistant and four starvation-sensitive RCCs by qRT-PCR analysis (Figure 1B). *TNFSF10* was significantly up-regulated in all starvation-resistant RCCs compared with sensitive RCCs. At the protein level, TRAIL up-regulation was generally confirmed in starvation-resistant RCCs, in which cell death was induced by inhibiting HIF2-alpha, compared with those of starvation-sensitive RCCs, with the exception of Caki1 (Figure 1C and 1D). These results suggested that *TNFSF10* may contribute to the mechanism of cell death caused by inhibiting HIF2-alpha in dormant-like/starvation-resistant RCC.

### HIF2-alpha and c-FLIP avert TRAIL apoptosis in starvation-resistant RCCs

**Figure 2:**
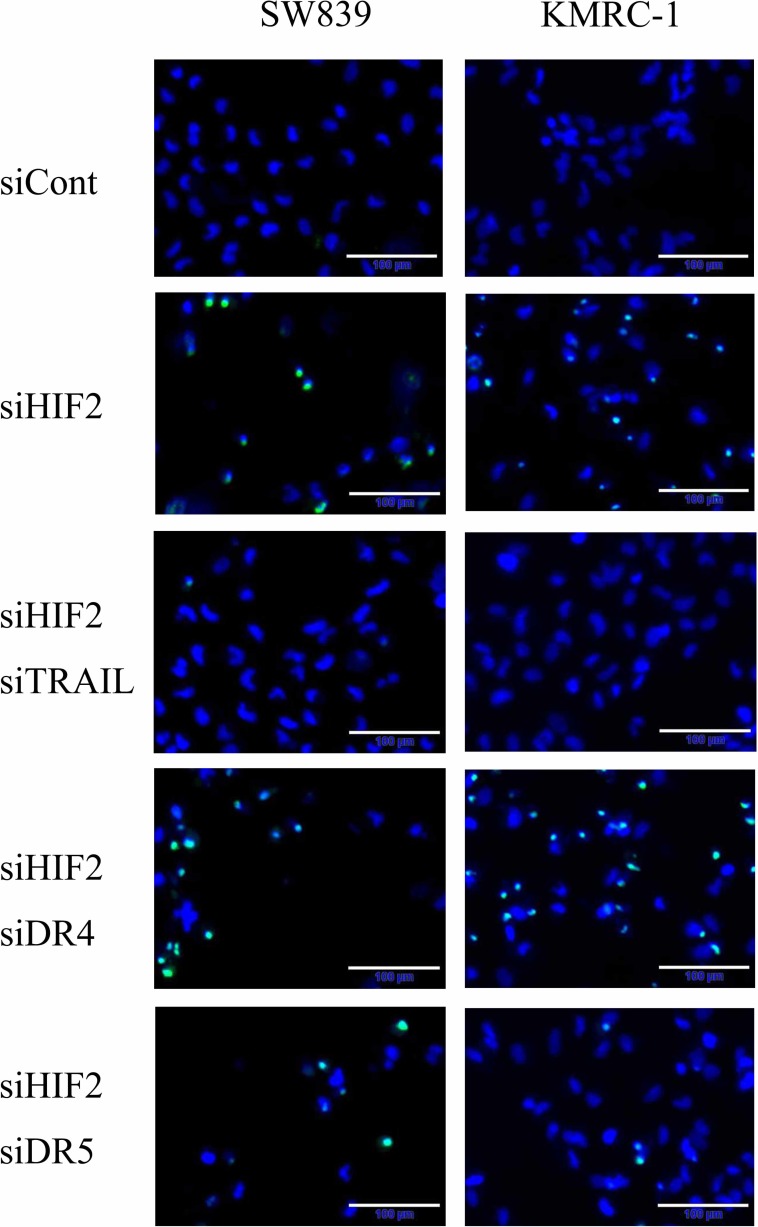
Analysis of apoptosis signals in starvation-resistant RCC Photographs of signals of CellEvent™ Caspase-3/7 Green Detection Reagent (apoptotic cells, green) and Hoechet33342 (nuclei of total cells, blue) in the starvation-resistant cells SW839 and KMRC-1 after 2-day treatment with the indicated siRNA reagents. Note that the apoptotic cell death induced by knockdown of HIF2-alpha (siHIF2) in starvation-resistant RCCs was averted by knockdowns of TRAIL (siTRAIL) and its receptor DR5 (siDR5).

**Figure 3:**
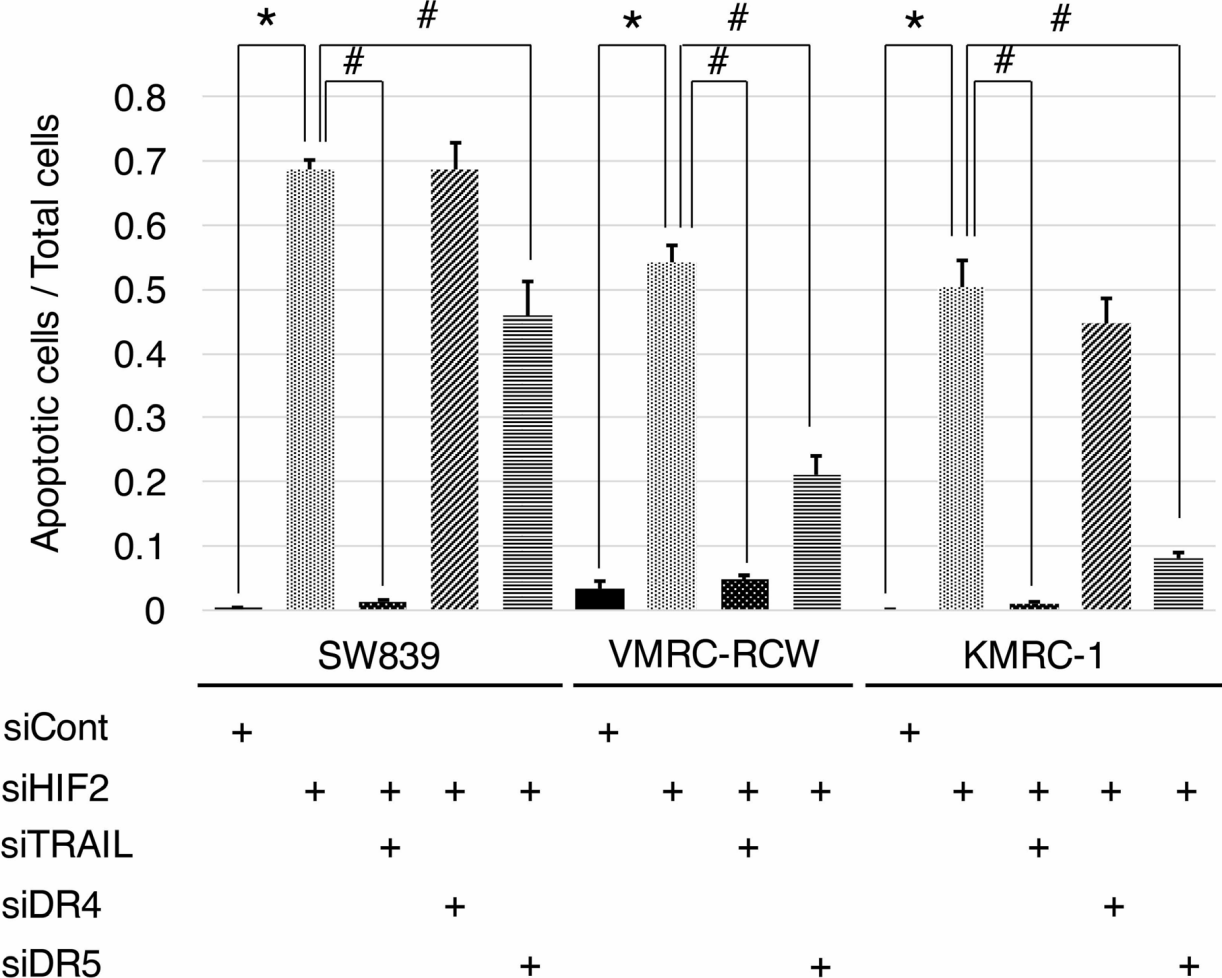
Graphic representation of apoptosis signals in starvation-resistant RCC cell lines Signals of CellEvent^™^ Caspase-3/7 Green Detection Reagent (apoptotic cells, green) and Hoechet33342 (nuclei of total cells, blue) shown in Figure 2 were quantified in all starvation-resistant RCC cell lines (SW839, VMRC-RCW, and KMRC-1). Error bars represent standard errors from six independent experiments. ANOVA: In SW839, F (4, 25) = 127.76, *p* = 2.955e^–16^; in VMRC-RCW, F (3, 20) = 120.46, *p* = 5.675e^–13^; in KMRC-1, F (4, 25) = 89.997, *p* = 1.818e^–14^; *p* < 0.05, pairwise comparisons using *t* tests with pooled SD vs. siCont (^*^) or siHIF2 (^#^), respectively. Note that apoptotic cell death induced by knockdown of HIF2-alpha (siHIF2) in starvation-resistant RCCs was signifcantly averted by knockdowns of TRAIL (siTRAIL) and its receptor DR5 (siDR5).

**Figure 4:**
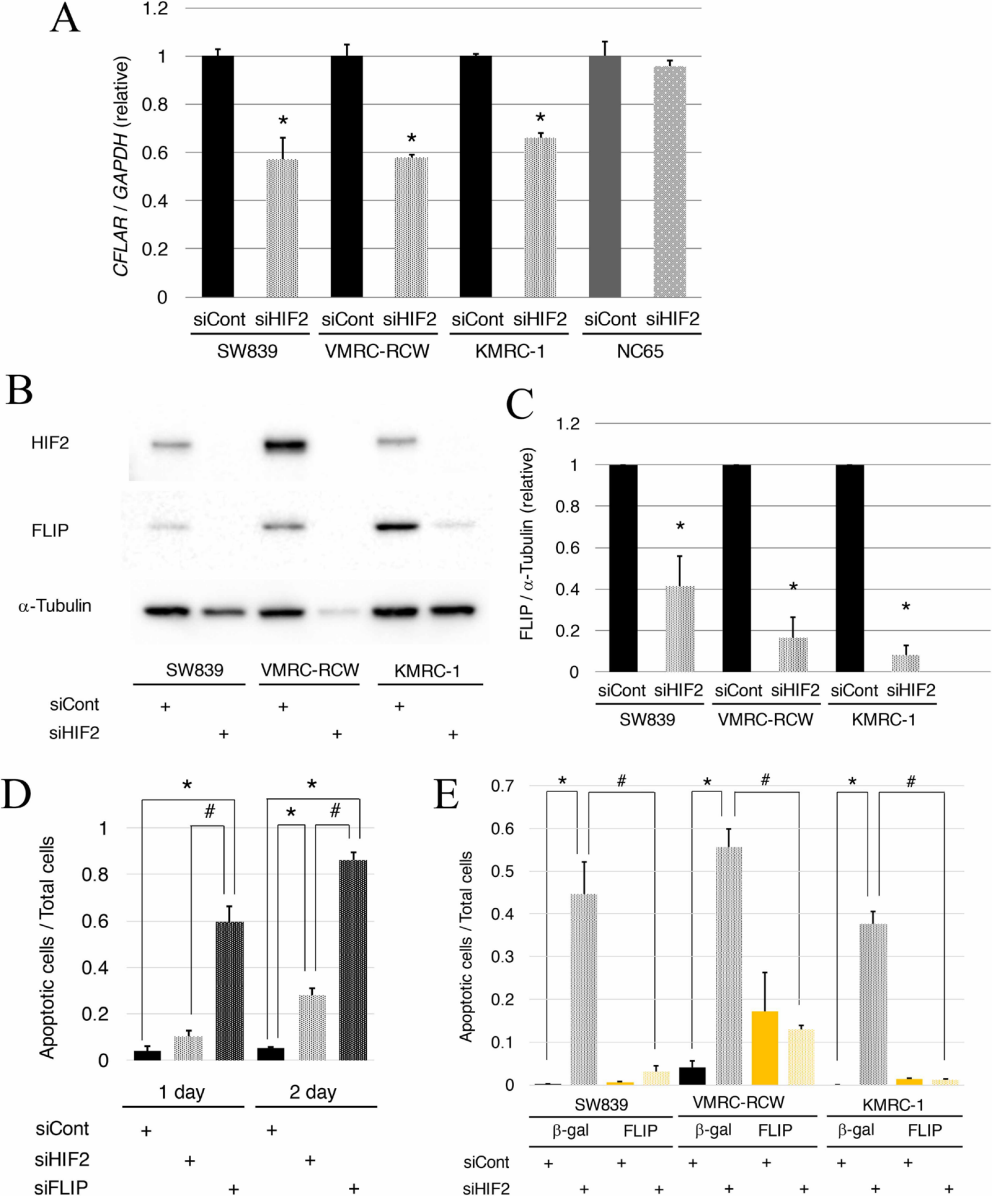
Reduction of c-FLIP following HIF2-alpha knockdown affects cell survival in starvation-resistant RCCs (**A**) Quantitative RT-PCR of c-FLIP (*CFLAR*) in starvation-resistant RCC cell lines (SW839, VMRC-RCW, and KMRC-1) and the starvation-sensitive cell line (NC65) after 2-day treatment with siHIF2. (**B**) Immunoblots of c-FLIP in all starvation-resistant RCC cells after 2-day treatment with siHIF2. (**C**) Graphic representation of data shown in panel B. Transcriptional (A) and protein expressions (B–C) were normalized against *GAPDH* and α-tubulin, respectively. Error bars represent standard errors from three independent experiments. Student’s *t*-test (two-tailed) was used to compare with each control (siCont). Asterisks indicate *p* < 0.05. (**D**) Quantified graph of signals of CellEvent^™^ Caspase-3/7 Green Detection Reagent (apoptotic cells, green) and Hoechet33342 (nuclei of total cells, blue) in the starvation-resistant SW839 RCC cell line under 1-day and 2-day treatments with the indicated siRNA reagents (siCont, siHIF2, and siFLIP). Error bars represent standard errors from six independent experiments. ANOVA: 1-day, F (2, 15) = 49.388, *p* = 2.514e^–7^; 2-day, F (2, 15) = 290.18, *p* = 1.023e^-12^; *p* < 0.05, pairwise comparisons using *t* tests with pooled SD vs. siCont (^*^) or siHIF2 (^#^), respectively. (**E**) Quantified graph of signals of CellEvent^™^ Caspase-3/7 Green Detection Reagent (apoptotic cells, green) and Hoechet33342 (nuclei of total cells, blue) in all starvation-resistant RCC cell lines, in which β-gal or c-FLIP was introduced lentivirally, after 2-day treatment with siHIF2. Error bars represent standard errors from five or six independent experiments. ANOVA: In SW839, F (3, 20) = 33.782, *p* = 5.047e^–8^; in VMRC-RCW, F (3, 20) = 20.269, *p* = 2.809e^–6^; in KMRC-1, F (3, 19) = 155.57, *p* = 1.506e^–13^; *p* < 0.05, pairwise comparisons using *t* tests with pooled SD vs. siCont (^*^) or siHIF2 (^#^), respectively, of the control cells, in which β-gal was introduced lentivirally. Note that knockdown of c-FLIP induced augmentation of apoptosis with knockdown of HIF2-alpha (D), and that artificial introduction of c-FLIP averted apoptosis completely in all starvation-resistant RCC cell lines (E).

**Figure 5:**
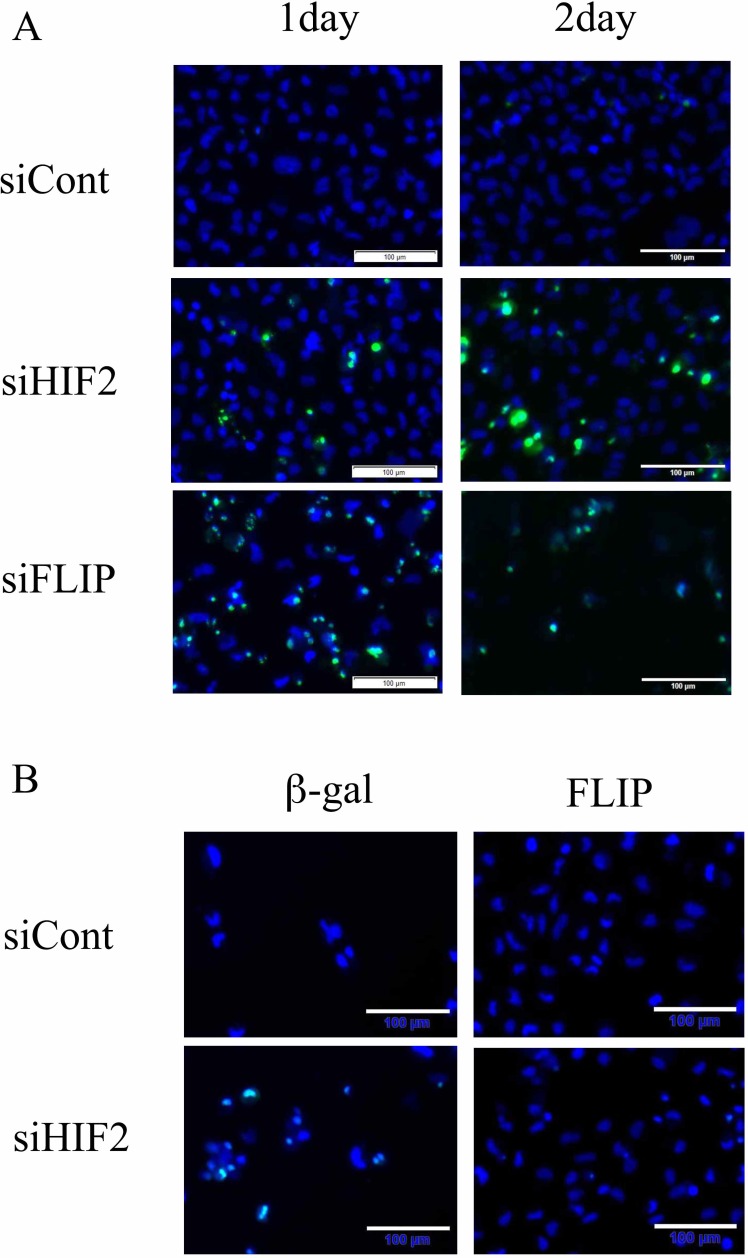
Knockdown of c-FLIP induces apoptosis and its introduction averts apoptotic cell death in the starvation-resistant RCC cell line SW839 (**A**) Apoptotic signals of CellEvent^™^ Caspase-3/7 Green Detection Reagent (apoptotic cells, green) and Hoechet33342 (nuclei of total cells, blue) in the starvation-resistant RCC cell line SW839 under 1-day and 2-day treatments with the indicated siRNA reagents (siCont, siHIF2 and siFLIP). (**B**) Photographic representation of SW839 cells, in which β-gal or c-FLIP was introduced lentivirally, after 2-day treatment with siHIF2. Note that knockdown of c-FLIP induced similar or greater apoptosis, and exogeneous introduction of c-FLIP completely averted apoptosis with siHIF2 in SW839 cells. These are photographic representations of data shown in Figure 4D and 4E.

To clarify the contribution of *TNFSF10* in the mechanism of cell death induced by knockdown of HIF2-alpha in dormant-like/starvation-resistant RCC, siRNA for *TNFSF10* (siTRAIL) was introduced into all three dormant-like/starvation-resistant RCCs, accompanied by siRNA for HIF2-alpha (siHIF2). In the co-introduction of siRNAs for *TNFSF10* (siTRAIL) and *EPAS1* (siHIF2), siTRAIL signifcantly averted apoptotic cell death induced by siHIF2 in dormant-like/starvation-resistant RCC (Figures 2 and 3). Knockdown of death receptor 5 (DR5), a death receptor for TRAIL [13], via introduction of siDR5 also reduced apoptotic cell death induced by siHIF2. However, silencing of death receptor 4 (DR4), another death receptor for TRAIL [14], did not produce the same result (Figures 2 and 3). Although the efficiencies of DR4 and DR5 knockdowns were similar, the differences between the biological effects of siDR4 and siDR5 may depend on each basal expression of them in starvation-resistant cell lines, because the RPKM value of DR5 was approximately 10-fold greater than that of DR4 in SW839 cells (Supplementary Figure 1). Therefore, apoptotic cell death related to TRAIL may be averted by HIF2-alpha in dormant-like/starvation-resistant RCCs. FADD-like apoptosis regulator, c-FLIP (*CFLAR*) is annotated as an inhibiting gene for TRAIL-induced apoptosis [15]. Expression of c-FLIP was reduced by knockdown of HIF2-alpha in three dormant-like/starvation-resistant RCC cell lines (Figure 4A–4C), whereas knockdown of c-FLIP (siFLIP) induced apoptotic cell death more rapidly and effectively than knockdown of HIF2-alpha in starvation-resistant RCC (Figures 4D and 5A). Overexpression of c-FLIP, introduced lentivirally into three dormant-like/starvation-resistant RCC cell lines, completely cancelled apoptotic cell death induced by siHIF2 (Figures 4E, 5B and Supplementary Figure 2). These data demonstrated that dormant-like/starvation-resistant RCCs evaded TRAIL-induced apoptotic cell death by HIF2-alpha activation followed by induction of c-FLIP.

### Abundance of TRAIL *(TNFSF10)* may be associated with poor prognosis in patients with metastatic RCC

**Figure 6:**
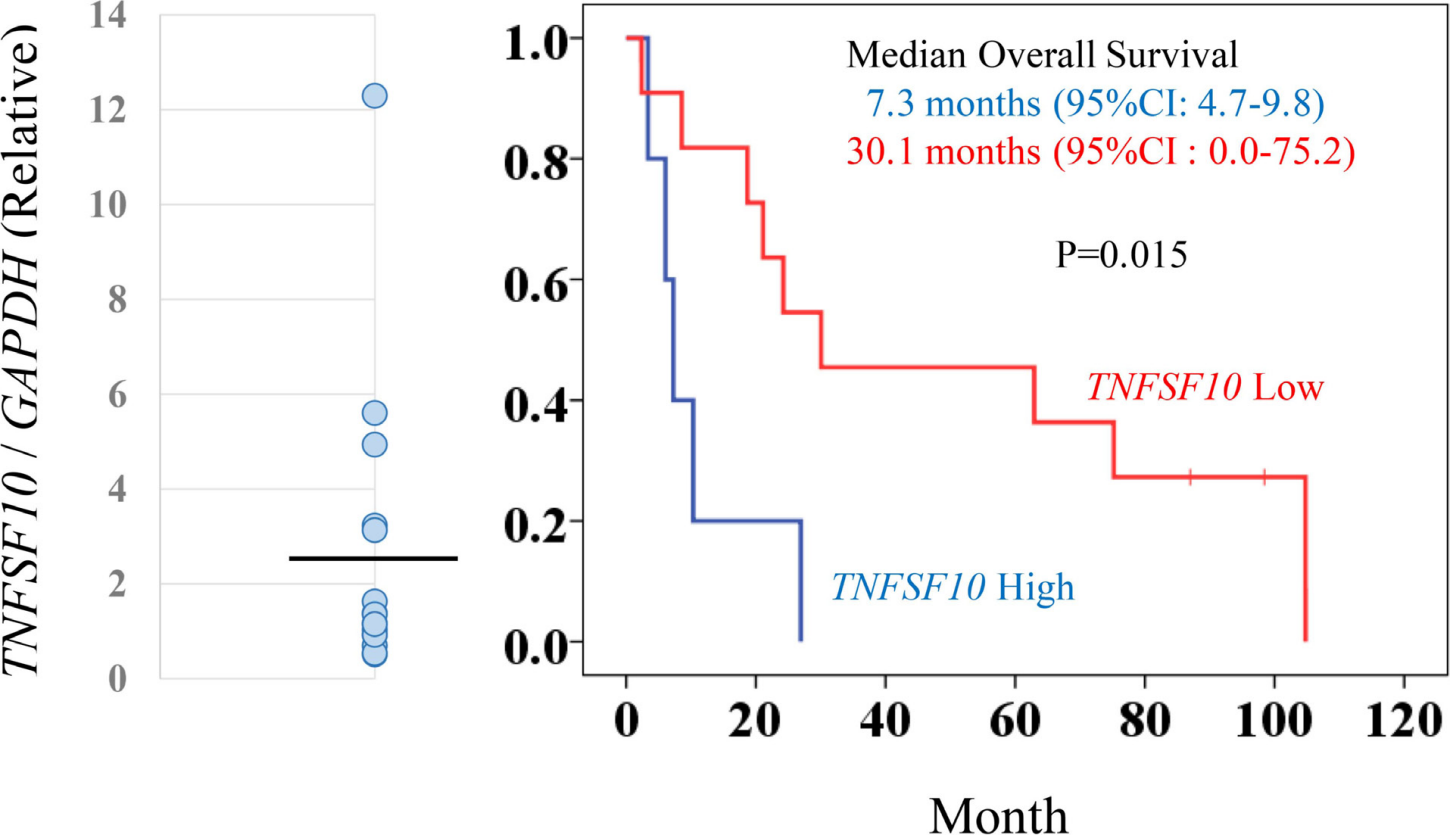
*TNFSF10* (TRAIL) expression levels associated with prognosis for patients with metastatic RCC To determine *TNFSF10* (TRAIL) expression levels, clinical samples from patients with metastatic RCCs were analyzed by qRT-PCR with *GAPDH* normalization (left panel). The black line indicates the average value of *TNFSF10* expression. The RCC patients were categorized in high or low *TNFSF10* expression groups based on the average value. Kaplan-Meier survival curves for *TNFSF10* in patients with metastatic RCC (right panel). The high *TNFSF10* group was associated with significantly shorter survival periods compared with the low *TNFSF10* group after chemotherapy (log-rank test, *p* = 0.015).

The role of TRAIL (*TNFSF10*) expression as a predictor of clinical outcome in patients with metastatic RCC treated with inhibitory agents for tyrosine kinase and/or mTOR in the Cancer Institute Hospital was investigated. The characteristics and demographic data of the 16 patients examined in the cohort are shown in Supplementary Table 2. These patients were classifed into two groups based on the average value of *TNFSF10* expression levels (Figure 6); patients with higher *TNFSF10* expression levels had significantly shorter survival periods than those with lower *TNFSF10* levels (7.3 vs. 30.1 months, respectively) (Figure 6). This comparative analysis suggested that patients with higher *TNFSF10* expression were resistant to commonly used agents targeting tyrosine kinase and mTOR.

## DISCUSSION

The present study showed that TRAIL-induced apoptosis may be evaded by HIF2-alpha activation followed by induction of c-FLIP in dormant-like/starvation-resistant RCC. Cells evaded apoptotic cell death with higher HIF2-alpha levels followed by abundant c-FLIP expression, despite TRAIL being highly expressed in these cells. Subsequently, the knockdown of HIF2-alpha reduced c-FLIP expression and induced apoptotic cell death due to abundant TRAIL. TRAIL-induced apoptotic cell death may be specific to dormant-like/starvation-resistant RCC, which co-expressed HIF2-alpha and TRAIL. Upregulation of c-FLIP has been found in various tumor types, and c-FLIP is an important target for cancer therapy using TRAIL [15]. Our immunohistochemical analysis revealed an association of c-FLIP up-regulation with shortly suvival periods in RCC patients (Supplementary Figure 3B–3C). This is the first study showing that the knockdown of HIF2-alpha reduced the expression of c-FLIP. In pancreatic cancer cells with abundant HIF2-alpha, the knockdown of HIF2-alpha induced apoptotic cell death via endogenous TRAIL, regulated by Survivin rather than c-FLIP [16]. Malignat cells driven by high levels of HIF2-alpha can evade apoptotic cell death triggered by TRAIL, with c-FLIP following sustainable HIF2-alpha. This evidence suggests that HIF2-alpha may be a potentially new therapeutic target for TRAIL-resistant cancer cells.

TRAIL was highly expressed in starvation-resistant RCC cells compared with starvation-sensitive RCC cells. Expression of TRAIL may be promoted by FOXO following the down-regulation of the PI3K-Akt signaling pathway. However, further investigation is warranted to confirm this hypothesis. Global transcriptional data of SW839, a typical starvation-resistant RCC cell line, indicated that “Class I PI3K signaling events mediated by Akt” was down-regulated, in contrast to the data of NC65, which was a typical starvation-sensitive cell [11]. The PI3K-Akt signaling can inhibit transcriptional activity of FOXO, and TRAIL is a target of “FOXO signaling pathway”, according to the database of Kyoto Encyclopedia of Genes and Genomes (KEGG). Inhibition of PI3K-Akt signaling may actively promote FOXO transcription followed by high expression of TRAIL in starvation-resistant RCC cells.

RCCs expressing high levels of TRAIL were associated with poor prognosis. Therefore, expression levels of TRAIL may become a predictive biomarker for the prognosis of clinical outcome in patients with metastatic RCCs. The present investigation was a pilot study involving a limited number of patients treated by various agents. Further clinical studies are warranted to confirm the prognostic value of TRAIL abundance. The findings presented herein were surprising, considering that TRAIL is essentially a ligand associated with apoptosis in cancer cells. The mode of action of abundant TRAIL in dormant-like/starvation-resistant RCCs remains unclear. TRAIL was detected in exosomes prepared from dormant-like/starvation-resistant RCCs (Supplementary Figure 3A). Exosomes were recently reported to contribute to progression of certain types of cancers [17]. Thus, TRAIL-bearing exosomes may contribute to the progression of disease. In the present immunohistochemical analysis (Supplementary Figure 3B), TRAIL was detected with infiltrating lymphocytes in all the tumors, although tumorous TRAIL was found in only 1 out of 30 cases of RCCs. Exosomal TRAIL was hardly detected using immunohistochemistry. Abundance of c-FLIP/CFALR in the tumor was found predominantly in the cases with short survival (Supplementary Figure 3B, lower row) rather than in the cases with longer survival (Supplementary Figure 3B, upper row), and this difference was statistically significant (Supplementary Figure 3C). Therefore, these data suggested that RCCs with poor prognosis may evate TRAIL-induced apoptosis through high expression of c-FLIP, and this may be due to sustainable HIF2-alpha expression.

**Figure 7:**
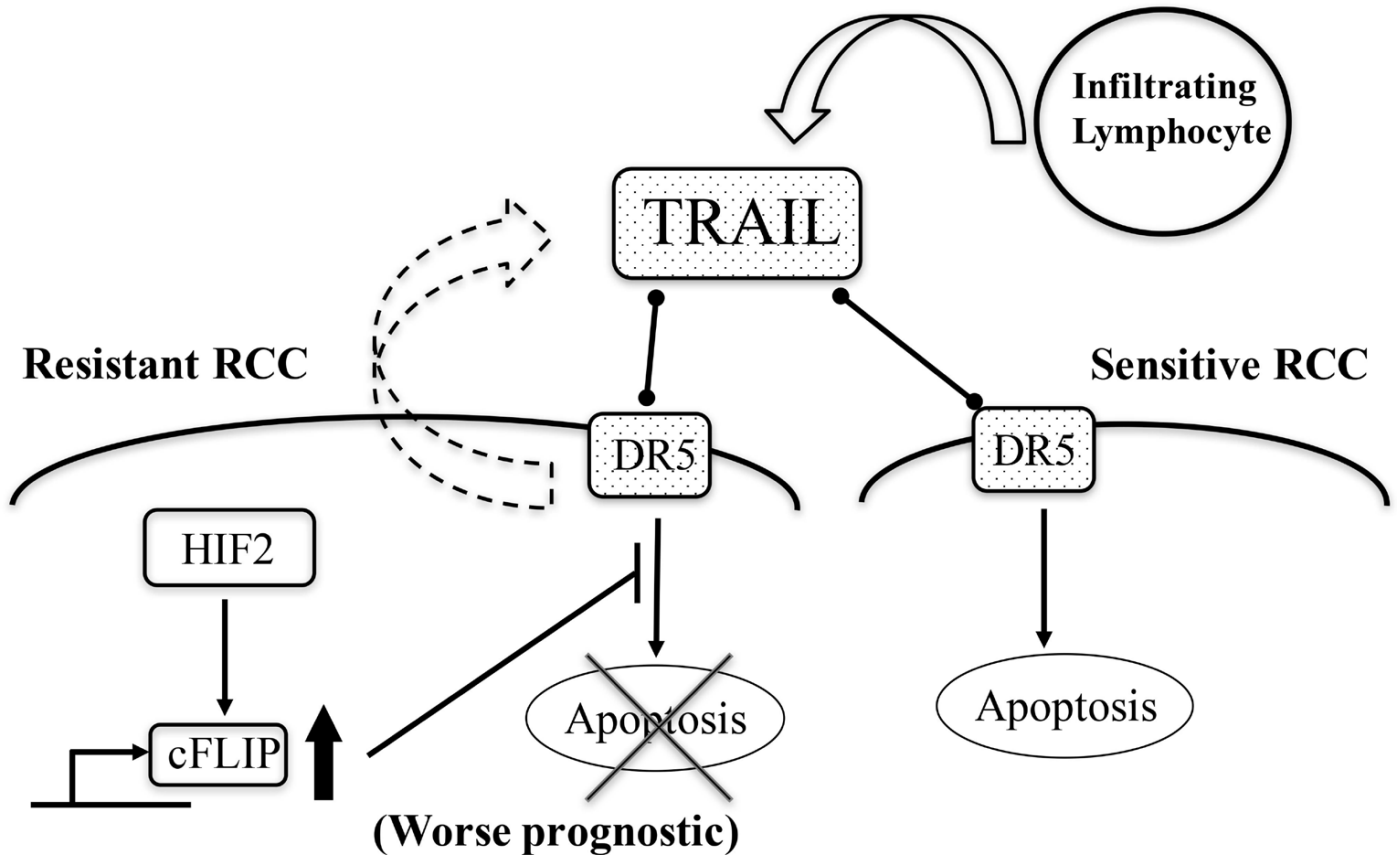
A schematic summary of all experimental results TRAIL abundance caused by infiltraring lymphocytes and exosomes from starvation-resistant RCC cells induce apoptosis into starvation-sensitive RCC cells; however, the dormant-like/resistant RCCs may evade TRAIL-induced apoptosis, with c-FLIP following sustainable expression of HIF2-alpha. Subsequently, abundance of TRAIL may foster the development of RCCs and affect malignancy and prognosis of RCCs. Thin arrows indicate activation of a target and events. The thick arrow pointing upwards signifies an increase in the levels of the corresponding molecules. The cross signifies inhibition of an event. The bar arrow indicates suppression of a target. Dot arrows indicate molecular interactions. Curved arrows indicate secretion of vesicles.

In conclusion, TRAIL-induce apoptosis was attenuated by c-FLIP following sustainable HIF2-alpha expression in dormant-like/starvation-resistant RCC. Abundance of TRAIL may foster the development of RCCs, and affect their malignancy and prognosis in RCCs (Figure 7).

## MATERIALS AND METHODS

### Cell lines and cell culture conditions

Three starvation-resistant RCC cell lines (SW839, VMRC-RCW and KMRC-1) and four starvation-resistant RCC cell lines (Caki1, Caki2, NC65 and ACHN) were used in this study. These cell lines were purchased from either the American Type Culture Collection, Riken Cell Bank, Cell Resource Center for Biomedical Research in Tohoku University (Sendai, Japan) or the Japanese Collection of Research Bioresources (Osaka, Japan). All cell lines were maintained in RPMI 1640 (Nakarai Tesque, Kyoto, Japan), containing 25 mM glucose, supplemented with 10% fetal calf serum, penicillin (100 U/ml) and streptomycin (100 μg/ml) at 37° C in a humidified 5% CO_2_ atmosphere.

### Patients

The medical records of patients with RCC, treated in the Cancer Institute Hospital (Japanese Foundation for Cancer Research, Tokyo, Japan) between 2008 and 2014, were retrospectively reviewed. In all patients, ccRCC was confirmed by pathological diagnosis. Histopathology was reviewed according to the 2004 World Health Organization classification [18]. This study was carried out in compliance with the Helsinki declaration, and was approved by the institutional review board of the Cancer Institute Hospital (2009–1029). Written informed consent was obtained from all patients in this study.

### Antibodies

The anti-TRAIL (#3219) and anti-HIF2-α (#7096) rabbit monoclonal antibodies were purchased from Cell Signaling Technology (Beverly, MA, USA). The anti-CD63 (ab134045) rabbit monoclonal antibody was purchased from Abcam (Cambridge, MA, USA). The anti-c-FLIP (sc-5276) mouse monoclonal antibody was purchased from Santa Cruz Biotechnology (Santa Cruz, CA, USA). The anti-α-tubulin (#T9026, DM1A) mouse monoclonal antibody was purchased from Sigma-Aldrich (St. Louis, MO, USA).

### Immunoblotting

Cells were lysed in Laemmli-SDS buffer, subjected to SDS-polyacrylamide gel electrophoresis, and electro-transferred to membrane filters (Immuno-Blot^®^ PVDF membranes, Bio-Rad Laboratories, Richmond, CA, USA). The filters were incubated overnight with a primary antibody in TBS-T buffer containing 2% bovine serum albumin (BSA) and incubated for 1 hour in horseradish peroxidase-conjugated anti-mouse or anti-rabbit secondary antibody (Cell Signaling Technology) diluted 1:5,000 in TBS-T buffer containing 2% BSA. Immunoreactivity was detected using the Luminata™ Classico Western HRP substrate (Millipore Corporation, Billerice, MA, USA) with LAS4000 (Fujifilm, Tokyo, Japan) and quantified using the MultiGauge™ software (Fujifilm) against anti-α-tubulin antibody as internal control.

### Analysis of apoptosis signals

Cells (1 × 10^4^)/100 μl were plated onto 96-well culture plates and cultured at 37° C. After treatment, 5 µM CellEvent™ Caspase-3/7 Green Detection Reagent (Thermo Fisher Scientific, Waltham, MA, USA) and 10 μg/ml Hoechet33342 (Dojindo, Mashiki, Kumamoto, Japan) were added to the treated cells and the plates were incubated for 30 minutes at room temperature. These cells were observed using a fluorescence microscope IX83 (Olympus, Tokyo, Japan) and analyzed using the imaging software cellSens (Olympus).

### Quantitative reverse transcription-polymerase chain reaction (qRT-PCR)

Total RNA was obtained from RCC cells and tissues of patients who had undergone radical nephrectomy using acid guanidinium thiocyanate-phenol-chloroform [19]. Quantitative RT-PCR was performed using the LightCycler^®^ 480 SYBG Master I Mix and LightCycler^®^ 480 System II (Roche Diagnostics, Mannheim, Germany). Gene expression was normalized against the *GAPDH* gene. Primer sequences are listed in Supplementary Table 3A. All quantification analyses were performed in triplicate.

### siRNA

RNA duplexes for siRNAs targeting human HIF2-alpha (*EPAS1*, s4699), TRAIL (*TNFSF10*, s16664), c-FLIP (*CFLAR*, s16864), DR4 (*TNFRSF10A*, s16764) and DR5 (*TNFRSF10B*, s16756) were purchased from Life Technologies. Scrambled control RNA duplexes (Silencer^®^ Select Negative Control #1 siRNA, 4390844) were also purchased from Life Technologies. Cells were transfected with RNA duplexes using Lipofectamine^®^ RNAiMAX reagents (Thermo Fisher Scientific) according to the manufacturer’s protocol.

### Preparation of lentiviruses

Lentiviral cDNA vectors for *CFLAR* were obtained from Dharmacon (GE Dharmacon, Cambridge, UK). pLenti6 vector containing β-gal cDNA (Invitrogen, Thermo, MA, USA) was used as overexpression control. Lentiviruses transferring cDNA were prepared using the Lenti-X^™^ HTX packaging system (Clontech, Takara Bio, Shiga, Japan) according to manufacturer’s instructions. SW839, VMRC-RCW and KMRC-1 cells transferred by >20 MOI of each lentivirus were selected in the presence of 10 μg/ml blasticidin, expanded and used in the experiments.

### Analysis of global transcriptional data

Global transcriptional data for starvation-resistant RCC cell line SW839 and for starvation-sensitive RCC cell line NC65 were acquired from the DDBJ under accession number DRA005074, PRJDB5127, and SAMD00058068-73 as described previously [11].

### Statistics

The data are reported as means ± standard error (SE). The values were derived from at least three replicate experiments. Statistical analyses were performed using the free software R. One-way factorial analysis of variance (ANOVA), accompanied by pairwise comparisons using *t* tests with pooled standard deviation (SD) was used to compare multiple group means. Student’s *t*-test (two-tail) was used to compare the means of two groups. A *p*-value of < 0.05 was considered to denote statistical significance. In the clinical study, overall survival periods were measured from the day of initial administration of the therapeutic agents until the day of disease-specific death. Time-to-event distributions were estimated using Kaplan-Meier curves and statistically analyzed (log-rank test) using the SPSS software (SPSS for Windows, version 17.0, SPSS Inc.).

## SUPPLEMENTARY MATERIALS FIGURES AND TABLES


