# Supplementary material for: Abundance of TRAIL attenuated by HIF2α and c-FLIP affects malignancy in renal cell carcinomas
Source: Oncotarget. 2018 May 1;9(33):23091–101. doi: 10.18632/oncotarget.25214 (PMC5955402; doi:10.18632/oncotarget.25214)
Supplement: Supplementary file 1 [file oncotarget-09-23091-s001.pdf]

Abundance of TRAIL attenuated by HIF2α and c-FLIP affects malignancy in renal cell carcinomas

SUPPLEMENTARY MATERIALS

Isolation of concentrated supernatants and exosomes

For the isolation of exosomes, semi-confluent RCCs were maintained in RPMI 1640 in 100-mm plates, cultured in OptiMEM (Thermo Fisher Scientific) for 3 days. Supernatants from culuture plates were collected using the CentriPlus 10 (Thermo Fisher Scientific), according to the manufacturer's protocol. Exosomes were isolated from the concentrated supernatants using the Exosome Isolation Kit Pan (Miltenyi Biotec, Bergisch Gladbach, Germany) according to manufacturer's protocol. The contents of TRAIL and CD63 in the extracted exosomes were evaluated by immunoblotting.

Patients for the immunohistochemical analysis

The medical records of 30 patients with RCC, treated at Shiga University of Medical Science Hospital (Shiga, Japan) between 2011 and 2016, were retrospectively reviewed. The mean age of the patients was 61.1 years (34–82 years). The patients included 24 males and 6 females. The mean follow-up period was 30.4 months (2–63 months) and the endpoint of the observation period was defined as death or last visit to the hospital. Histopathology was reviewed according to the 2004 World Health Organization classification [19]. This study was carried out in compliance with the Helsinki declaration, and was approved by the Ethics Committee of Shiga University of Medical Science (No. 27–192). Written informed consent was obtained from all patients in this study.

Immunohistochemistry

Surgical specimens were transferred to 10% buffered formalin and fixed overnight. Fixed samples were embedded in paraffin and serially sliced into 5-µm sections. After dewaxing, sections were autoclaved at 120° C for 1 min in 10 mM sodium citrate buffer (pH 6.0) and immersed in 0.3% H_2O_2. Subsequently, they were then incubated overnight at 4° C with a primary antibody against TRAIL/TNFSF10 (diluted 1:500, #3219, Cell Signaling Technology, Beverly, MA) or c-FLIP/CFLAR (diluted 1:500, #6G11A6, GeneTex, Irvine, CA). Sections were rinsed with phosphate-buffered saline and incubated with a secondary antibody conjugated with horseradish peroxidase (Simple Stain MAX-PO, Nichirei, Tokyo, Japan) at room temperature for 1 hour. Sections were subsequently stained with 3.3′-diaminobenzidine tetrahydrochloride and counter-stained with hematoxylin.

Microscopic evaluation

The expression of TRAIL and c-FLIP in each RCC was immunohistochemically determined by relative comparison to the expressions of normal proximal renal tubule as an internal control on the same slide. TRAIL fractions were detected with infiltrating lymphocytes in all RCC tissues, and expression of tumorous TRAIL was immunohistochemically shown in only 1 out of 30 cases. Expression of c-FLIP was detected in all the RCC cases, and two qualitative grades for specimens were defined according to the intensity of c-FLIP expression, namely i) in "c-FLIP-positive" samples, the staining intensity of the tumor was similar or greater than that of the normal proximal renal tubule; and ii) in "c-FLIP-negative" samples, the staining intensity of the tumor was less than that of the normal proximal renal tubule (Supplementary Figure 3B). The correlation between c-FLIP positivity and survival period was evaluated using Fisher's exact test. A p-value of < 0.05 denoted statistical significance using the SPSS software (SPSS for Windows, version 17.0, SPSS Inc.).

Supplementary Table 1: Summary of differences in starvation-sensitive and starvation-resistant types of renal cell carcinomas

	Sensitive type	Resistant type
Cell lines	NC65, ACHN, Caki1, Caki2	SW839, VMRC-RCW, KMRC-1
Glucose Deprivation	Cell death	Survival
N-$GlcNAc_2$	Produced	No
Cell cycle	G2/M arrest	Unchanged
Mitochondrial quality	Low	High
Lipid and sugar stock	Few	Abundant
Hydroxyl-HIF2-alpha	No	Produced
siHIF2-alpha	No effect	Cell death

Supplementary Table 2: Characteristics of metastatic renal cell carcinoma patients treated with molecular targeting agents

	TRAIL high (n = 5)	TRAIL low (n = 11)	p value
**Gender**			0.37
Male	4	11	
Female	1	0	
**Median age (range)**	71 (56–77)	64 (51–75)	0.30
**Performance status**			
0 or 1	5	11	
2 or greater	0	0	
**Presence of metastasis at diagnosis**			0.17
negative	0	2	
positive	5	9	
**Pathological diagnosis**			
clear cell	5	11	
non-clear cell	0	0	
**Nephrectomy**			
negative	0	0	
positive	5	11	
**Sarcomatoid component**			0.11
negative	2	10	
positive	3	1	
**MSKCC classification**			0.64
Intermediate	4	10	
Poor	1	1	

Supplementary Table 3: Oligonucleotides used for qRT-PCR

Gene symbol	Forward (5′ to 3′)	Reverse (5′ to 3′)
CFLAR	AGTACAAGCAGTCTGTTCAAGGAG	GATTCCTGAATGGATTTCTTCACTG
TNFSF10	CGAGCTGAAGCAGATGCAGGAC	GAGTTGCCACTTGACTTGCCAG
GAPDH	GGGAGCCAAAAGGGTCATCATC	TGGCATGGACTGTGGTCATGAG

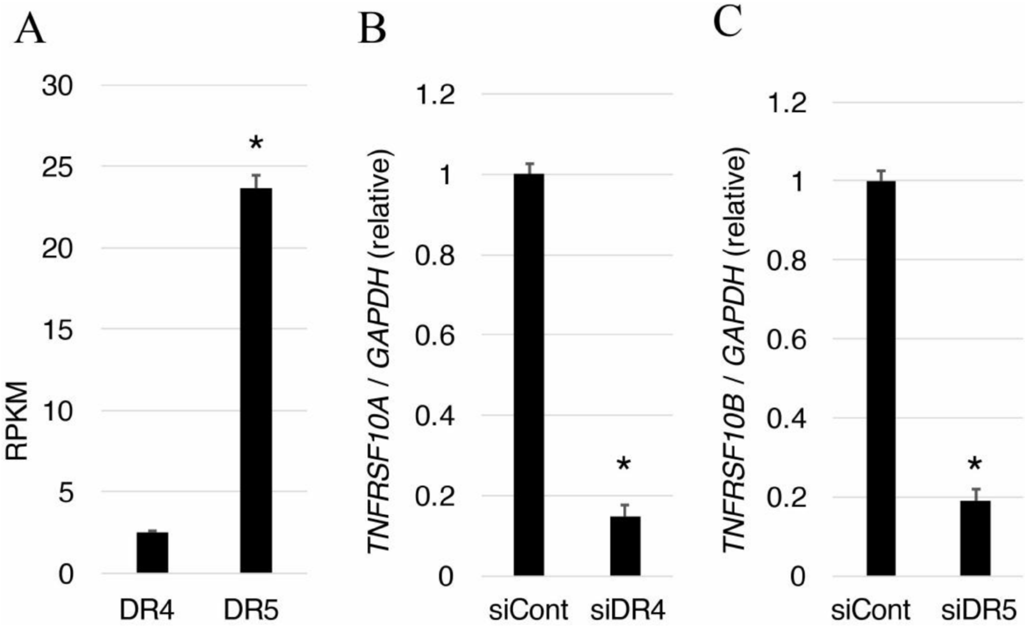

Supplementary Figure 1: Abundant expression of DR5 compared with DR4 in the starvation-resistant RCC cell line SW839. (**A**) RPKM values of *TNFRSF10A* (DR4) and *TNFRSF10B* (DR5) in the starvation-resistant RCC cell line SW839. Globally transcriptional data indicated that DR5 was approximately 10-fold more abundant than DR4. (**B**) Quantitative RT-PCR of *TNFRSF10A* in RCC cell lines SW839 after 2-day treatment with siDR4. (**C**) Quantitative RT-PCR of *TNFRSF10B* in RCC cell lines SW839 after 2-day treatment with siDR5. Student's *t* test (two-tailed) was used to compare with each control (siCont). Asterisks indicate $p < 0.05$.

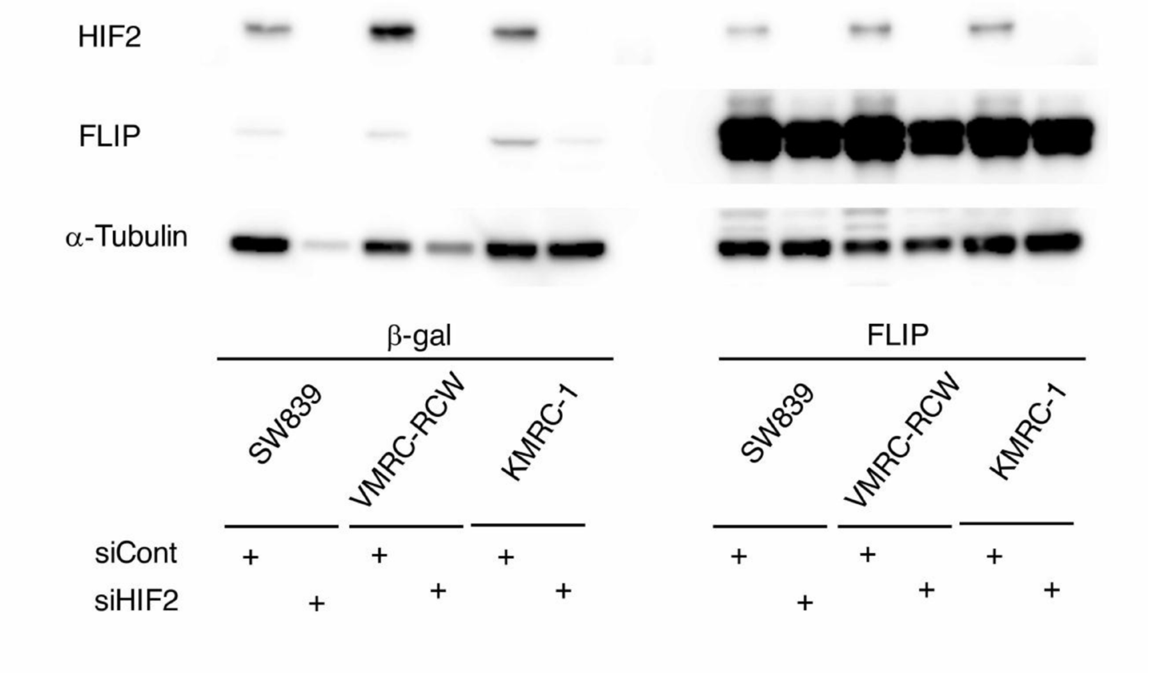

Supplementary Figure 2: Immunoblots of HIF2 and c-FLIP in the starvation-resistant RCC cell lines in which β-gal or c-FLIP was introduced lentivirally, after 2-day treatment with siHIF2. After 2-day treatment with siHIF2, imunoblots were conducted in the starvation-resistant RCC cell lines, in which β-gal or c-FLIP was introduced lentivirally. Note that the starvation-resistant RCC cell lines in which c-FLIP was introduced, expressed c-FLIP abundantly after 2-day treatment with siHIF2.

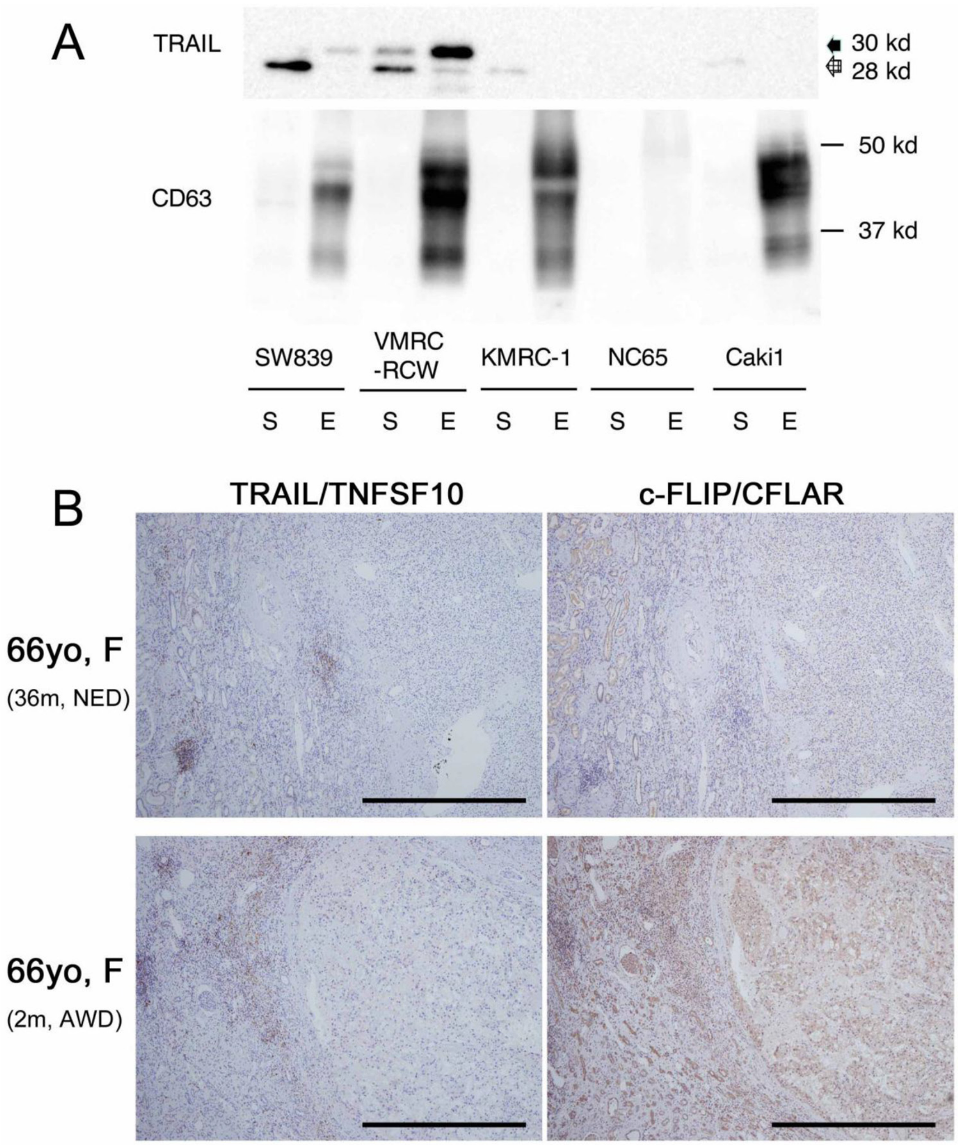

C

	c-FLIP/CFLAR	
	+	-
Survival (m)		
＜24	8	4
24≦	3	15

Fisher's exact test, p=0.0086

Supplementary Figure 3: Detective evaluation of TRAIL and c-FLIP in RCCs. (A) Detection of TRAIL in the exosome fractions of RCCs. CD63 was used as the exosome marker. "S" and "E" show the concentrated superrnatants and exosomes, respectively. Note that TRAIL-including exosomes were abundantly detected in the starvation-resistant RCC cell lines SW839 and VMRC-RCW. (B) Immunohistochemically representative samples of TRAIL and c-FLIP in RCCs. In the upper and lower rows, longer and shorter disease-specific survival cases are respectively demonstrated. Bars indicate 1 mm. The left and right half of each photograph respectively include normal tissues composed of tubulus & glomerulus and malignant tissues of clear cell RCC. Note that TRAIL was detected in infiltrating lymphocytes in both cases, and c-FLIP was detected in normal tubular and glomerular tissues. Although c-FLIP was subtle in the clear cell RCC tissues of the upper case, it was quite abundant in the tumors of lower case, which indicated shorter disease-specific survival. (C) The correlation between clinically survival periods and immunohistochemically c-FLIP positivity was statistically evaluated using Fisher's exact test. Note that c-FLIP positivity was observed predominantly in shorter survival cases ($p = 0.0086$).
